# Detection of Prostate Carcinoma in an Asymptomatic Individual Initiated by an Immunological Biopsy—A Case Report

**DOI:** 10.32604/or.2025.068555

**Published:** 2026-01-19

**Authors:** Simon Burg, Audrey Laure Céline Grust

**Affiliations:** 1 Department of Oral and Maxillofacial Surgery, University Medical Center Hamburg-Eppendorf (UKE), Martinistr. 52, Hamburg, 20246, Germany; 2 Department of Oral and Maxillofacial Surgery, Division of “Regenerative Orofacial Medicine”, University Medical Center Hamburg-Eppendorf (UKE), Martinistr. 52, Hamburg, 20246, Germany

**Keywords:** Epitope detection in monocytes, apoptoic-associated cell population 10 (Apo10), transketolase-like protein 1 (TKTL1), case report

## Abstract

**Background:**

With a total of 1.46 million new cases and 396,792 deaths in 2022, prostate cancer is a major medical challenge around the world. Detecting and treating cancer at earlier, preferably localized stages can significantly increase survival rates. Here, a novel blood-based cancer screening as a pre-test in combination with targeted MRI imaging enabled the early diagnosis of prostate cancer.

**Case Description:**

We present the case of a 64-year-old man who participated in a prospective, interventional, multicenter cancer screening study where an immunological biopsy-based technique served as a part of a novel screening technique. This immunology technique represents a blood test exploiting two biomarkers, which may allow for the identification of individuals at an early stage of tumor development. Due to the elevated biomarker levels of Transketolase-like protein 1 (TKTL1) and Apoptoic-associated cell population 10 (Apo10), magnetic resonance imaging (MRI) was indicated for further clarification. A multiparametric MRI of the pelvis/prostate revealed an enlarged prostate gland and several suspicious lesions classified as Prostate Imaging Reporting and Data System (PI-RADS) 4 and PI-RADS 5. In further assessments, both lesions were categorized as an acinar adenocarcinoma of the prostate (Gleason Score 6, International Society of Urological Pathology (ISUP) 1, no perineural infiltration). After surgical resection, the tumor was classified histopathologically as an adenocarcinoma, pT2c pN0 (0/7), L0, V0, Pn1, R0, Gleason score 7a, ISUP 2.

**Conclusions:**

The combination of the TKTL1/Apo10 blood test and subsequent imaging made it possible to detect a developing prostate carcinoma in a localized stage. All in all, this case report proves not just the ability but also the potential of the TKTL1/Apo10 blood test for early detection of (pre-)malignant lesions, which still present with a promising prospect for a cure.

## 1 Introduction

With a worldwide incidence of 14.2%, prostate cancer was the second most commonly diagnosed cancer in men in 2022 [1]. With 396,792 deaths in 2022, it is still the fifth leading cause of cancer death in men worldwide [1]. With the introduction of screening procedures and improved treatment, prostate cancer mortality has declined in many countries [2,3]. Detecting and treating cancer at earlier, preferably localized stages can significantly increase survival rates [4]. Prostate-specific antigen (PSA) testing may have contributed to this, but there is no clear evidence to date [4], as elevated PSA levels can be caused by conditions other than prostate cancer, such as inflammation or benign prostatic hyperplasia [2]. Nevertheless, efforts have been made to increase the specificity of PSA testing, for instance, with photoelectrochemical (PEC) immunoassays for point-of-care (POC) testing, allowing for PSA detection in the range of 0.01–50 ng/mL [5,6], and it is still considered a valuable addition for prostate cancer diagnostics. PEC aptasensors have even been shown to selectively detect PSA at values as low as 0.52 pg/mL with high reproducibility, thus matching, if not even outstanding PSA ELISA kits [5,7]. Furthermore, a urine based POC test using enzyme-encapsulated protein engineered metal-organic framework-derived biominerals allows for fast and easy screening of prostate cancer individuals by direct detection of sarcosine in urine samples, which can be used for disease monitoring as well [8]. It is still of great importance to further develop efficient and cost-effective diagnostic methods that allow the detection of a wide variety of tumor types in early, preferably asymptomatic stages [9,10].

One current development in the early detection of cancer is the PanTum Detect^®^ blood test. This test is based on the unique technique of epitope detection in monocytes (EDIM). Activated monocytes/macrophages (CD14+/CD16+), which belong to the group of circulating cancer-associated macrophage-like cells (CAMLs), phagocytose tumor cells and contain intracellular tumor proteins [11–13]. This method was first successfully applied to measure the PSA level in circulating macrophages about two decades ago [14–16]. Thereby, increased levels of circulating PSA containing macrophages were detected in patients with prostate cancer compared to patients with benign conditions [14–16]. Moreover, Herwig et al. [16] compared the intra- and extracellular measured PSA levels of patients with benign and malignant disease. While no significant differences in extracellular PSA levels were observed, elevated PSA levels were detected in circulating PSA-positive macrophages from patients with prostate malignancies. The authors suggested that macrophages phagocytize prostate cancer cells via a target-oriented immune response and thus contain tumor material intracellularly when they return to the bloodstream. This represents the rationale for the observed high specificity of the test. In addition, intracellular PSA staining was significantly different in localized and metastatic prostate cancer, showing its great potential as a new noninvasive diagnostic tool [16]. The correlation between the presence of a tumor and the level of cancer-related epitopes in circulating macrophages was also shown in a study by Todenhöfer et al. [17]. After radical prostatectomy, a significant decrease in elevated Apo10 and TKTL1 in macrophages was detected. Therefore, the detection of Apo10 and TKTL1 in pre- and postsurgical blood samples could be used to monitor the surgical removal of tumors. Furthermore, the increase in Apo10 and TKTL1 offers a new option for the early detection of recurrence [18].

The PanTum Detect^®^ blood test exploits the biomarkers Apoptoic-associated cell population 10 (Apo10) and Transketolase-like protein 1 (TKTL1), which are related to two independent and fundamental biophysical mechanisms that are altered during tumorigenesis. Apo10, an epitope of deoxyribonuclease X (DNaseX), enables the detection of a DNaseX protein variant in the nucleus (Apo10) concomitant with inhibited endonuclease activity and inhibited apoptosis [11]. TKTL1 controls cell cycle and synthesis of ribose, the essential building block for DNA synthesis and DNA repair [19,20]. In addition, TKTL1 allows the conversion of glucose to acetyl-CoA [21,22] and lactic acid [23–25]. Both metabolites play a crucial role in malignancy, since acetylation and lactylation of histones activate tumor-promoting genes as well as induce immunosuppression [26–28]. Recently, it has been demonstrated that lactic acid secreted by a cancer cell is able to activate tumor-promoting genes and immunosuppression in neighboring cells, thereby creating cancer cells in a mutation-independent and epigenetically driven mechanism [27]. TKTL1 is the biochemical basis of the “Warburg effect”, an anaerobic glucose fermentation to lactic acid even in the presence of oxygen (aerobic glycolysis) [23–25,29]. In addition to the above-described epigenetic role in malignant transformation, lactic acid permits an acid-based matrix degradation of the surrounding tissue, leading to invasive growth, metastasis [30], and immunosuppression of tumors [26–28]. The metabolic role of TKTL1 is in line with the fact that activation of the anaerobic glucose fermentation in the presence of oxygen (“Warburg effect”/aerobic glycolysis) is one of the earliest events in the activation of oncogenes in somatic cells [23,31].

The biomarkers Apo10 and TKTL1 can be analyzed via flow cytometry from peripheral blood samples using specific antibodies. The epitope detection in monocytes (EDIM) technology has already been applied in several studies [32–34], showing its great potential in immunological biopsy-based screening.

The EDIM-TKTL1/Apo10 blood test was Conformité Européenne (CE) certified as PanTum Detect^®^ in 2017 in Germany.

The cut-off value in the PanTum Detect^®^ test system is crucial for achieving diagnostic sensitivity and specificity, and the optimal balance between these two parameters. The combined score is a dimensionless number and consists of the two individual scores for Apo10 and TKTL-1 and counts as positive when it reaches or exceeds the threshold of 260, with an Apo10 score of ≥140. This cut-off value has been investigated and established in previous studies [33,35,36].

Based on these findings, the advantages of the test include its ease of use, cost-effectiveness, minimal invasiveness, and high specificity. Disadvantages include the lack of data on the test’s use in monitoring the progression of cancer, and the fact that the test may be temporarily inapplicable due to certain immunomodulatory medications, injuries, or infections.

In clinical practice, multiparametric magnetic resonance imaging (mpMRI) is the method of choice for a detailed anatomic assessment of the prostate with superior soft tissue resolution [37]. Thereby, the sensitivity and positive predictive value of MRI are increased by combining anatomic (transverse relaxation time (T)1-weighted and multiplanar T2-weighted images) and functional sequences (diffusion-weighted imaging with apparent diffusion coefficient maps and dynamic contrast-enhanced imaging) [38]. The introduction and update of the Prostate Imaging Reporting and Data System (PI-RADS) has enabled the standardization of image acquisition and interpretation with the goal of detecting clinically significant prostate cancers [39,40].

**Figure 1:**
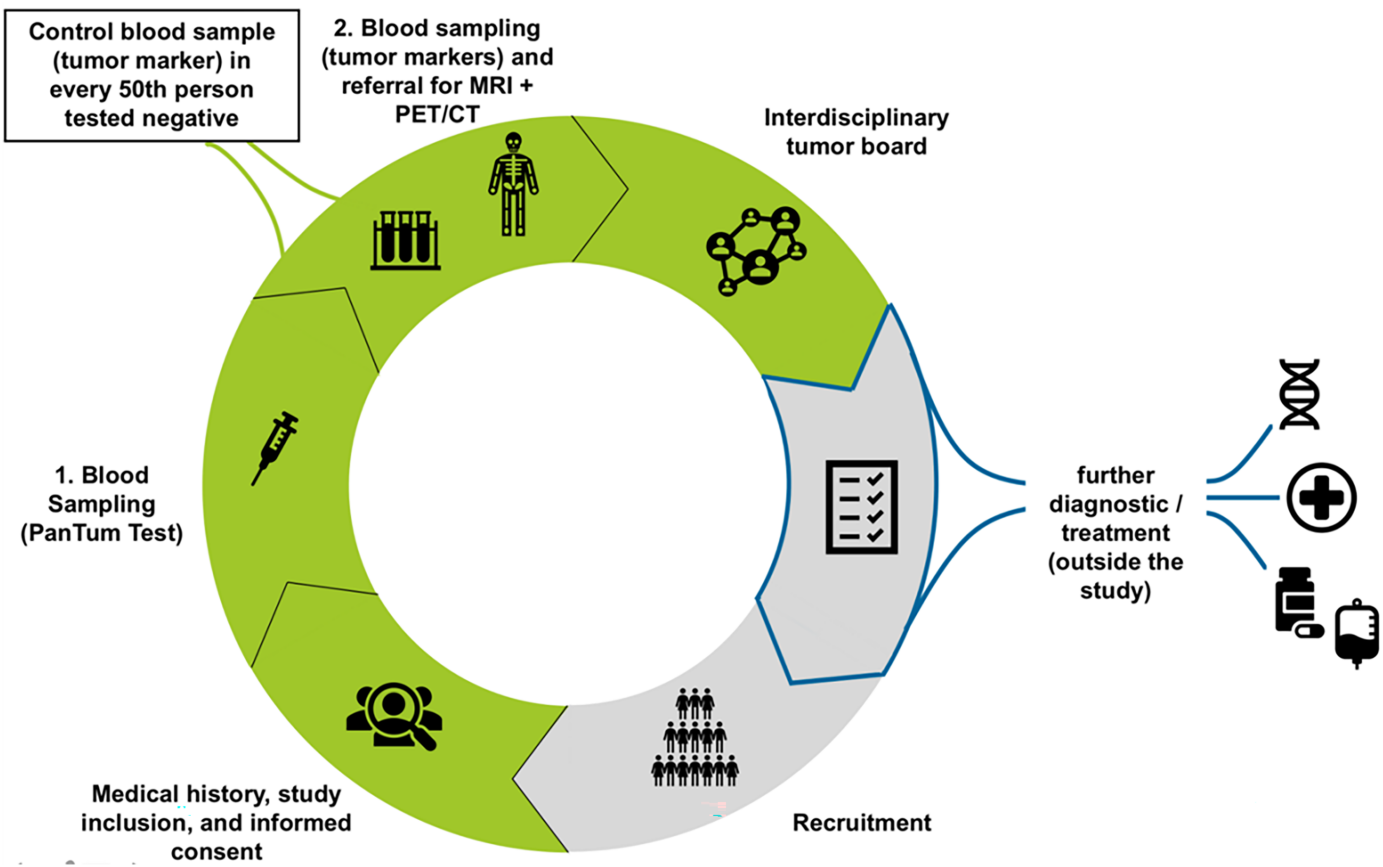
Work flow for the PanTum Detect® study. Initially, blood sampling for the PanTum Detect® Test is performed. In case of a positive test value (Total Score ≥260 and Apo10 value ≥140), a second blood draw is indicated for a tumor marker panel evaluation (CEA, Ca 19-9, AFP, PSA, Ca 125, Ca 15-3, ß-HCG, CRP). Afterwards, patients are referred for further radiological imaging according to study protocol (FDG-PET/CT and MRI or only 3T pelvic MRI in case of elevated PSA levels). Following the required diagnostics, an interdisciplinary tumor board evaluates all findings to determine if/what further diagnostic/therapeutic measures may be required and refers the patient to specialist care (outside the study). CEA, Carcinoembryonic Antigen; Ca 19-9, Carbohydrate Antigen 19-9; AFP, Alpha-Fetoprotein; PSA, Prostate-Specific Antigen; Ca 125, Carbohydrate Antigen 125; Ca 15-3, Carbohydrate Antigen 15-3; ß-HCG, Beta-Human Chorionic Gonadotropin; CRP, C-Reactive Protein; FDG-PET/CT, Fluorodeoxyglucose Positron Emission Tomography/Computed Tomography; MRI, Magnetic Resonance Imaging; 3T pelvic MRI, 3-Tesla Pelvic Magnetic Resonance Imaging

Here, we present a case in which an asymptomatic patient was screened using the PanTum Detect^®^ blood test (see Fig. 1 for a brief description of the PanTum Detect^®^ Study). Due to the positive PanTum Detect^®^ test result, mpMRI was selected. This enabled the detection of a prostate tumor at an early and localized stage. The patient was completely cured through surgical resection. Additional chemotherapy or radiotherapy, with subsequent loss of quality of life, was not required.

In our opinion, improved early detection of cancer represents the most effective approach to curing cancer minimally invasively and as completely as possible. This not only reduces morbidity and mortality but could also help reduce public health costs. The combination of the TKTL1/Apo10 blood test and subsequent imaging appears to be suitable to fulfill this goal.

## 2 Materials and Methods

At least 2 mL of EDTA whole blood was collected after a minimum of 60 min of fasting using a Sarstedt Monovette (Sarstedt AG& Co. KG, Nümbrecht, North Rhine-Westphalia, Germany). Shipping to the laboratory was undertaken by a transport service provider specialized in shipping blood samples (Trans-o-flex GmbH & Co. KGaA, Weinheim, Baden-Württemberg, Germany), and the samples were stored at room temperature (18°C–25°C). Staining was performed with antibodies CD14 (OFC-14D), CD16 (Hi-16a), Apo10 (clone JFC19X63) (InVivo BioTech Services GmbH, Henningsdorf, Brandenburg, Germany), and TKTL1 (clone JFC12T10) (InVivo BioTech Services GmbH, Henningsdorf, Brandenburg, Germany). The dilution ratios for each antibody are adjusted based on the previous batch (i.e., Reference measurement) and based on BD (BD Biosciences, Heidelberg, Baden-Württemberg, Germany) bead control material. Flow cytometry analysis took place within 36 h after blood collection at PreMed Labor GmbH, Pfungstadt, Hesse, Germany, with a BD FACSCantoTM II Flow Cytometry (BD Biosciences, Heidelberg, Baden-Wuerttemberg, Germany) operating software BD FACSDivaTM software version 8.0.3 and version 9.0.1 (BD Biosciences, Heidelberg, Baden-Wuerttemberg, Germany). The test result is presented in the form of the PanTum Detect^®^ test score, which is a combination of Biomarker TKTL1 and APO10. Based on the PanTum Detect^®^, the test is either positive or negative. Test results were considered positive if the sum of the two individual scores for Apo10 and TKTL1 reached or exceeded the threshold of 260 and, in addition, the Apo10 individual score was ≥140. The abdominal MRI examination was performed at a radiological practice (“dieRadiologen”, Weiterstadt, Hesse, Germany) on a GE MR750 (3T) device, General Electric Healthcare, Chicago, IL, USA. To minimize intestinal motility, 40 mg of Buscopan (A. Natermann & Cie. GmbH, Subsidiary of Sanofi-Aventis Germany GmbH, Frankfurt/Main, Hesse, Germany) was administered intravenously before imaging. The examinations were performed natively and after intravenous administration of 20 mL of Dotarem contrast agent (Guerbert GmbH, Sulzbach, Hesse, Germany). The following sequences were created: T2 axial single shot 5 mm 2–3-blocks; T2 coronal FS propeller, T2 sagittal single shot; T1 axial 5 mm 2–3-blocks; axial DWI 2 blocks.

This study included human subjects who participated in the prospective, interventional, multicenter study “PanTum Detect^®^” (ZYA-IVD-20202) at the University Hospital Hamburg-Eppendorf and the Precura Center Darmstadt. The consenting assessment of the Ethics Committee of the Hamburg Medical Association required under Section 20 (1) Sentence 1 of the Medical Devices Act was granted on 01 June 2021 (ethics approval number: 2020-10377-MPG-ff). The handwritten informed consent was obtained from the patient. Besides, this study was prepared according to the CARE case report guideline, and a CARE checklist was provided [41]. Please see Supplementary Material for more details.

## 3 Case Report

This case presents a 64-year-old male who participated in the prospective, interventional, multicenter study “PanTum Detect^®^” (ZYA-IVD-20202) and was further treated at the Sankt Katharinen Hospital in Frankfurt am Main. In this study, 5.114 asymptomatic individuals aged 50 to 70 years who had no personal history of cancer within the last eight years were enrolled and screened with the so-called PanTum Detect^®^ blood test. The handwritten informed consent was obtained from the patient.

A 64-year-old male, 191 cm tall, weighing 110 kg (Body Mass Index [BMI] 30.15 kg/m^2^), with neither own nor familiar history of cancer, has been suffering from hypertension, elevated cholesterol, and type 2 diabetes mellitus since 1991 (CCI-Score 3). Since then, he has been medicated with 50 mg Spironolactone (Ratiopharm GmbH, Ulm, Baden-Wuerttemberg, Germany), 160 mg Valsartan (Novartis AG, Basel, Switzerland), 1.5 mg Indapamid (Heumann Pharma GmbH & Co. Generica KG, Nuremberg, Bavaria, Germany) and, 40 mg Atorvastatin (Pfizer AG, New York City, NY, USA) once a day as well as with 5 mg Amlodipin (Pfizer AG, New York City, New York, USA) and 1000 mg Metformin (Merck KGaA, Darmstadt, Hesse, Germany) twice a day. He reported no further complaints or symptoms.

The PanTum Detect^®^ blood test revealed an elevated combined PanTum Detect^®^ score of 263, with a TKTL-1 score of 122 and an Apo10 score of 141. The subsequently determined prostate-specific antigen (PSA) value within the tumormarker panel was elevated at 4.29 ng/mL, while all other tested tumor markers (carcinoembryonic antigen [CEA], carbohydrate antigen 19-9 [CA 19-9], alpha-fetoprotein (AFP), beta-human chorionic gonadotropin [beta-hCG], c-reactive protein [CRP]) were not. Due to the elevated PanTum Detect^®^ score, imaging was required as part of the study for further clarification; according to the study protocol, mpMRI was performed because of the elevated PSA value. A specific 3T multiparametric MRI (GE Nr 750 (3Z), General Electric, 02210 Boston, MA, USA) of the pelvis and prostate was performed natively and after intravenous contrast administration.

**Figure 2:**
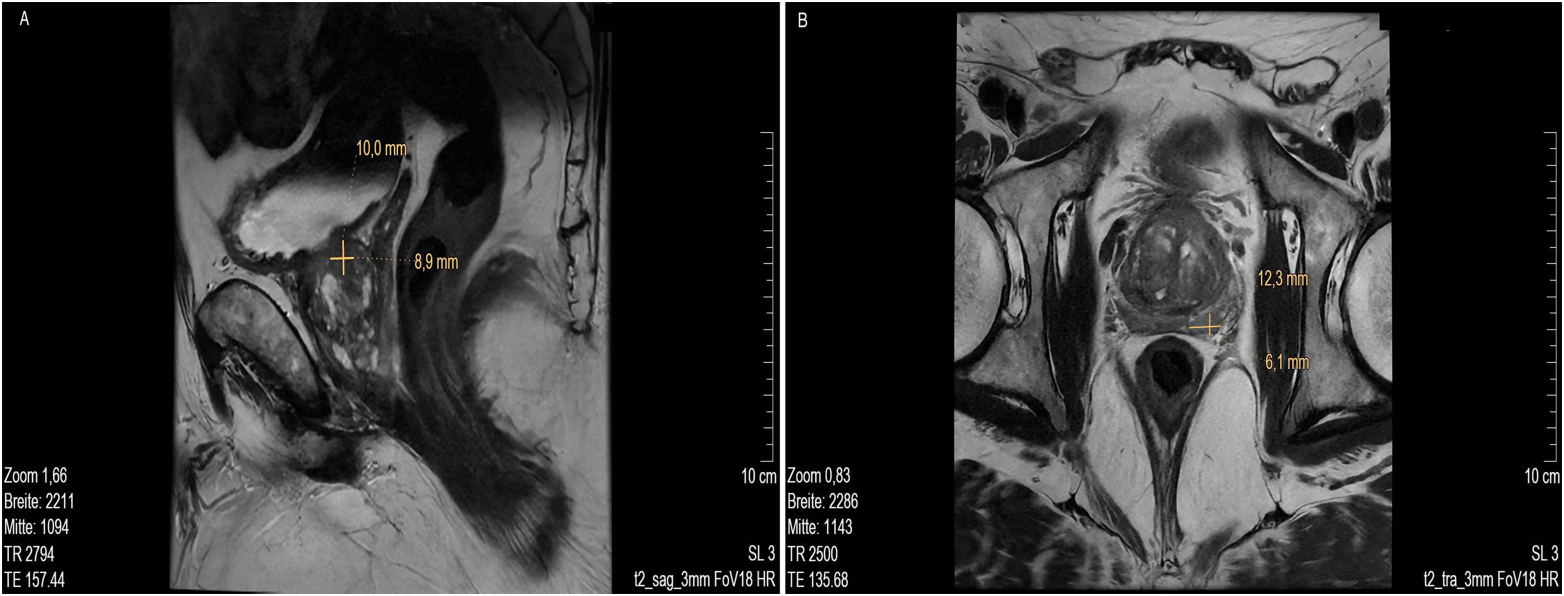
MRI scan lower pelvis (**A**) MRI sag T2 3 mm FOV 18, showing lesion 1 at about 9- to 10-o’clock position (marked with cross). (**B**) MRI low signal in T2 tra 3 mm FOV 18 with lesion 2 at approx. 5- to 6-o’clock position in the peripheral zone, close to the capsule, about 11 mm in length (marked with cross). MR images courtesy of Dr. med. Stefan Oehm from die Radiologen, Weiterstadt, Germany. MRI, Magnetic Resonance Imaging; sag, sagittal; FOV, field of view; tra, transverse

**Figure 3:**
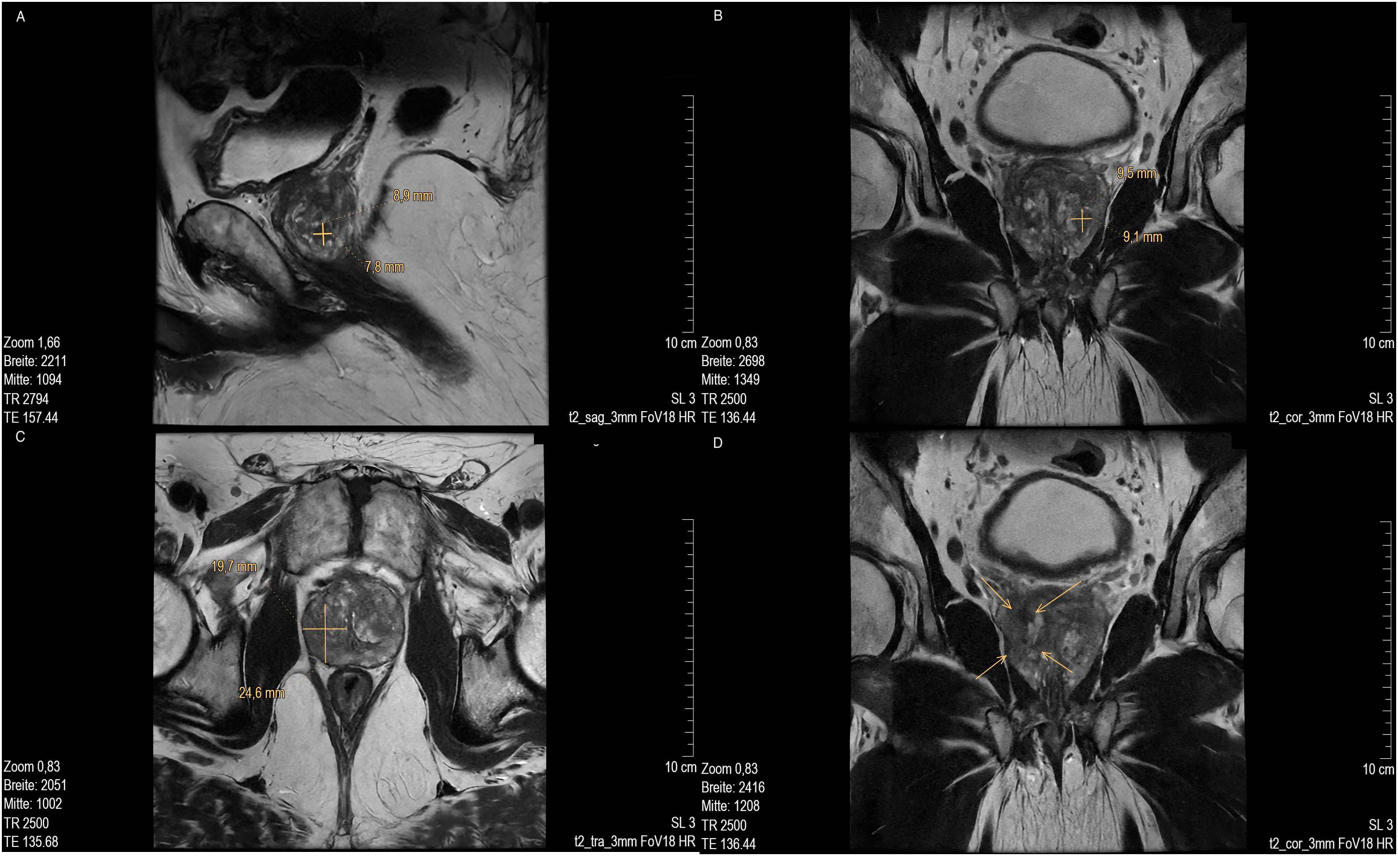
MRI scan of prostate of lesion 3. (**A**) Low in signal in T2 sag 3 mm FOV 18 and (marked with cross); (**B**) T2 cor 3 mm FOV 18. MRI of lesion 4 (marked with cross); (**C**) T2 tra 3 mm FOV18 (marked with cross) and (**D**) Low signal in T2 cor 3 mm FOV18 (marked with arrow). MR images courtesy of Dr. med. Stefan Oehm from die Radiologen, Weiterstadt, Germany. MRI, Magnetic Resonance Imaging; sag, sagittal; FOV, field of view; cor, coronal; tra, transverse

MR imaging revealed several suspicious lesions (Figs. 2 and 3) in the prostate gland and a wall thickening of the urinary bladder.

MRI showed an enlarged prostate gland (53 mm× 52 mm× 37 mm) depicted in Fig. 2A. A stromatous as well as adenomatous benign prostatic hypertrophy was visible. Lesion 1 is at a 9- to 10-o’clock position (marked with an arrow), located in the transitional/central zone, approximately 10 mm in size, without sharply defined edges and with a low signal in T2 sagittal (sag) 3 mm field of view (FOV) 18. The diffusion weighted imaging (DWI) displayed signal rich increasing b-values. The apparent diffusion coefficient (ADC) showed a low signal. In the perfusion study, increased perfusion with post initial contrast increase and wash-out was observed. Lesion 2 was located at approximately. 5- to 6-o’clock position (Fig. 2B, marked with an arrow) in the peripheral zone, close to the capsule, and about 11 mm in length (marked with a bar). Low signals were detected in T2 transverse (tra) 3 mm FOV 18. The lesion presented as planar, smoothly limited with a broad capsule contact. As for Lesion 1, Lesion 2 also displayed a rich signal in the DWI with increasing b-values, and a low signal in the ADC. No pathological accumulation of contrast agent was detectable. At approximately the 4 o’clock position, lesion 3 (marked with an arrow) was discernible in the transition zone with a length of ~10 mm. Low signal in T2 (Fig. 3A,B). Again, a rich signal was detectable in the DWI with increasing b-values, as well as a low signal in the ADC. In the perfusion study, post initial rapid enhancement with washout was observed. Lesion 4 is likely to correspond to the index lesion (area in Fig. 3C about 620 mm^2^, marked by arrows). A two-dimensional signal lowering from the transitional/peripheral zone to the central zone on the right side at 6- to 11-o’clock (Fig. 3C) was observed. This area also included large portions of the contralateral left peripheral zone, which was low in signal. The diagnostic finding showed a blurred border and a wide capsular contact, especially in the right circumference. In T2 (Fig. 3C,D) and the DWI, a low signal could be seen. The ADC also showed a flat low signal. No increased perfusion was detected. Based on the MRI data, lesions 2 and 3 were classified as PI-RADS 4, whereas lesions 1 and 4 were classified as PI-RADS 5 due to their morphology.

A pathologic anatomical assessment of the overall 24 punch biopsies of lesion 1 (biopsy 1–15) and 2 biopsy 16–24) was performed. Microscopy (punches 1–18, and 20–24) showed sporadically nodular glandular fields with papillary light cellular proliferates and a broad partly cell-rich stroma. In part, nodular confined cell-rich stromal areas were also present. Furthermore, no suspicious glandular formations or dysplastic epithelial changes were found in the punches. In contrast, punch biopsy no. 19 showed infiltrates of atypical glandular epithelia, often with enlarged nuclei and prominent nucleoli. There was no residual basal cell layer visible. The lumen exhibited partially atypical secretion products, with a cell-poor fibromuscular stroma present in between. Immunohistochemistry showed aberrant expression of alpha-Methylacyl-CoA Racemase (AMACR) and loss of p63-positive basal cells. Based on these data, the pathologic-anatomic diagnosis revealed the presence of an acinar adenocarcinoma of the prostate, Gleason Score 6 = 3 + 3, ISUP 1.

As a result, a laparoscopic transperitoneal robot–assisted radical prostatic vesiculectomy with bilateral nerve preservation and obturator lymphadenectomy was performed. Finally, the tumor was classified as adenocarcinoma of the prostate gland pT2c pN0 (0/7), L0, V0, Pn1, R0, Gleason score 7a = 3 + 4, ISUP 2.

The discrepancy between the initial Gleason score from the biopsy (3 + 3 = 6) and the improved score after prostatectomy (3 + 4 = 7a) can be attributed to several factors. First, prostate biopsies only sample a small portion of the gland, which may miss more aggressive cancer areas due to tumor heterogeneity. As a result, the biopsy may only reflect the low-grade portion. In contrast, the entire prostate is available for examination after surgery, allowing pathologists to more comprehensively assess tumor grade and detect higher-grade features that were not present in the biopsy specimens. Furthermore, differences in interpretation between pathologists—particularly because prostatectomy specimens are often reviewed by specialists—may lead to changes in grading. Finally, technical factors such as improved tissue processing and deeper sections in prostatectomy specimens further improve the detection of more aggressive disease. Together, these factors explain why an improvement in the Gleason score after surgical removal of the prostate is relatively common and expected.

The postoperative recovery was without complications, and further therapy was not necessary.

## 4 Discussion

Here, we present an approach in which an immunological biopsy-based technique, combined with subsequent imaging, demonstrated its notable potential to identify a malignancy at a localized stage in otherwise asymptomatic individuals. Just as the number of oncological clinical trials that include biomarkers has grown from 15% in 2000 to 55% in 2018, the number of clinical trials with pan-tumor biomarkers has increased substantially, reflecting the rising interest in biomarker-based cancer detection [42–44]. The blood test used here, PanTum Detect^®^, screens for the presence of the biomarkers Apo10/DNaseX and TKTL1, which are associated with two different fundamental biophysical processes in tumor cells [11,20–23,45,46] and accordingly may be indicative of the presence of various tumor types, including those for which there are no screening programs to date [1,10,11,47,48]. In recent years, liquid biopsy methods have been developed with a variety of analytes for tumor detection, such as circulating tumor DNA, cell-free DNA or RNA, circulating tumor cells, proteins, and metabolites [10,49,50]. Thereby, pan-cancer detection emerged as a promising, efficient, minimally invasive, cost and time-saving screening approach improving patient care and population health [9,10,51,52]. For example, in combination with PET-CT, the CancerSEEK blood test in the DETECT-A study was able to detect 16 entities by analyzing tumor-specific mutations in cell-free DNA and by protein biomarkers, followed by subsequent PET-CT when indicated [53]. Other tests, e.g., PanSeer and Galleri, are based on DNA methylation signature analysis and have been shown to detect 5 and over 50 cancer types, respectively [54,55]. But despite the great potential of next-generation sequencing-based tests to detect multiple types of cancer, there are still some challenges to overcome. The analysis of gene sequences is based on data matching from large databases and requires continuous machine learning using artificial intelligence. Another limitation could be the sample size, since the probed DNA circulates diluted in the bloodstream.

A major advantage of the PanTum Detect^®^blood test, besides its ease of use, is the fact that it is based on the EDIM technology. This enables immunological biopsy by utilizing the innate immune system’s ability to phagocytose and eliminate pre-malignant and malignant cells from throughout the body, including solid tissue types, by macrophages [11,15,16]. This method was already successfully applied to measure the PSA level of patients with histologically confirmed disease [14,15,17]. The PanTum Detect^®^test analyzes whole blood samples by flow cytometry for the presence of the two biomarkers TKTL1 and Apo10/DNaseX. This also allows the detection of premalignant or malignant cells that are not easily accessible through the skin or mucosa, as in the present case. Furthermore, in contrast to other blood tests that detect biomarker concentrations directly in the blood, this offers the advantage that the biomarkers are not detected diluted in the blood but concentrated in the cell volumes. The test’s high specificity stems from the innate immune system’s targeted phagocytosis. Due to antibody-dependent cellular phagocytosis (ADCP) of cancer cells, macrophages contain intracellular tumor material when they return to the bloodstream [12,13,56]. In other words, only cells that the immune system deems worthy of elimination are phagocytosed, which in turn has a positive influence on specificity. Limitations of this approach include the fact that the blood test is currently investigated exclusively in early cancer detection, but not for monitoring, staging, or follow-up. In addition, the use of certain medications that impair the immune system, as well as acute injuries, viral infections, and inflammatory diseases, may be temporary exclusion criteria, as they may influence blood test results. Furthermore, several still very small lesions may lead to an elevated PanTum Detect^®^ blood test value, although these may not be detectable yet with current imaging techniques due to their insufficient size and may also not lead to significantly elevated tumor marker values. However, if these small lesions continue to grow and the test is performed regularly, they will be detectable on imaging in time.

A further limitation to the presented cancer screening regime is the lack of longitudinal data and experiences with the use of the blood test in a large cohort. In addition, validation of the test’s predictive value and the potential for false positives/negatives is difficult to achieve because a control group is not feasible, and a gold standard does not exist.

Nevertheless, the experiences described here are encouraging, and due to its non-invasiveness and cost-effectiveness, the test could be used as a routine screening for large segments of the population. Existing screening programs (breast cancer, skin cancer, colon cancer, cervical cancer, prostate cancer) are primarily based on morphological criteria and could be supplemented with the PanTum Detect^®^Test to detect the presence of altered cell metabolism (TKTL1) or altered cell division (Apo10), thus making it more accurate.

The PanTum Detect^®^test could be used regularly (e.g., annually), and if abnormalities are detected, specific diagnostics (as in the case report presented here) could be initiated. The advantage would be that resource-intensive and invasive examination methods could only be used in a targeted manner if a tumor is already suspected through the preliminary test. Furthermore, the pantum test can screen for a wide variety of different tumors through a single blood sample, including tumors for which no screening program currently exists.

All in all, this could reduce complications from unnecessary invasive examinations while simultaneously detecting cancer at earlier, and therefore more curable, stages. This could potentially lead to a reduction in morbidity and mortality due to cancer.

## 5 Conclusion

As the second most common cancer type in men, prostate cancer is still one of the leading causes of cancer death in men worldwide [1]. Although surgical treatments and drug therapies have improved and expanded a lot in the last decades, many patients still have to undergo surgery followed by chemotherapy, immunotherapy, and/or radiation therapy, which represent a great physical and mental burden. It is therefore of the utmost importance to not only reduce the amount of cancer deaths but also to detect prostate cancer as early as possible to increase survival rates and improve the quality of life of the patients. In the case presented here, the combination of the TKTL1/Apo10 blood test and subsequent imaging made it possible to detect a developing prostate carcinoma in a localized stage, which could be removed completely by surgery alone. All in all, this case report proves not just the ability but also the potential of the TKTL1/Apo10 blood test for early detection of (pre-)malignant lesions, which still present with a promising prospect for a cure. Additional studies are required to further investigate the blood test and the presented screening regime.

## Supplementary Materials



## Data Availability

Derived data supporting the findings of this study are available from the corresponding author, Audrey Laure Céline Grust, on request.

## Supplementary Materials

The supplementary material is available online at https://www.techscience.com/doi/10.32604/or.2025.068555/s1.
